# Age-Related Differences in Mental Health Status at Psychiatric Discharge Among Patients Across 10 Acute Care Facilities in Alberta, Canada: Cross-Sectional Study

**DOI:** 10.2196/92360

**Published:** 2026-11-09

**Authors:** Wanying Mao, Ernest Owusu, Reham Shalaby, Hossam Eldin Elgendy, Belinda Agyapong, Ejemai Eboreime, Peter H Silverstone, Pierre Chue, Wesley Vuong, Arto Ohinmaa, Andrew J Greenshaw, Vincent I O Agyapong

**Affiliations:** 1 AI for Mental Health Program, Department of Psychiatry, St. Michael’s Hospital, Unity Health Toronto, Toronto, ON, Canada; 2 Department of Psychiatry, University of Alberta, Edmonton, AB, Canada; 3 Department of Psychiatry, Dalhousie University, 5909 Veterans Memorial Lane, Abbie J. Lane Memorial Building QEII Health Sciences Centre, Halifax, NS, B3H 2E2, Canada, 1 780-215-7771, 1 902-473-4887; 4 Clinical Operations Informatics Office, Health Shared Services, Edmonton, AB, Canada; 5 School of Public Health, University of Alberta, Edmonyon, AB, Canada

**Keywords:** mental health, digital mental health, Text4Support, psychiatric care, aging

## Abstract

**Background:**

The transition from psychiatric inpatient care to community settings is associated with heightened vulnerability and increased risk of adverse mental health outcomes. Understanding age-related differences in mental health status at discharge may inform the targeting of postdischarge interventions and potentially improve long-term outcomes.

**Objective:**

This study examined the associations between age and baseline demographic, clinical, and mental health characteristics among participants enrolled in the Text4Support program following discharge from psychiatric inpatient care.

**Methods:**

Baseline data collected between March 8, 2022, and February 29, 2024, from 1106 participants discharged from inpatient psychiatric units across Alberta, Canada, were analyzed. Participants were grouped by age (18‐25, 26‐40, and >40 years). Mental health outcomes were assessed using validated self-report measures. Chi-square tests and 1-way ANOVA or Welch ANOVA were used to examine age-related differences in categorized and continuous outcomes, respectively.

**Results:**

Significant age-related differences were observed across several mental health outcomes. Younger participants (18‐25 years) reported higher levels of depression, anxiety, and suicidal ideation, along with lower resilience and recovery scores compared with participants aged older than 40 years. Differences in quality of life were also observed, with younger adults reporting greater anxiety/depression and participants aged older than 40 years reporting more mobility-related problems.

**Conclusions:**

Marked age-related disparities in mental health status were evident at the point of psychiatric discharge, with younger adults demonstrating greater vulnerability. These findings underscore the importance of age-informed risk stratification and tailored transitional care strategies following inpatient psychiatric treatment and suggest that different strategies may be required depending upon age.

## Introduction

The transition from psychiatric inpatient care to community-based mental health services represents a particularly vulnerable period for individuals experiencing mental health conditions [1-3]. This phase is frequently associated with adverse clinical outcomes, including symptom exacerbation, relapse, hospital readmission, and an increased mortality risk [1-4]. Substantial evidence indicates that patients discharged from psychiatric facilities encounter significant challenges in the early postdischarge period, reflecting unmet clinical needs and limited continuity of care [1-4]. Understanding the clinical characteristics of patients at the point of discharge is therefore essential for informing targeted interventions and improving postdischarge outcomes.

Age is a key demographic factor that has been shown to influence the presentation, severity, and course of mental health conditions across the lifespan [5,6]. Younger and older adults often differ markedly in symptom profiles, coping strategies, service use patterns, and treatment engagement [6]. For example, younger adults may present with higher levels of affective symptoms and psychosocial stressors, whereas older adults may experience greater functional impairment, comorbidity, and barriers to accessing mental health care [6-8]. Despite these differences, age-related variations in mental health symptom burden at the point of psychiatric discharge remain underexplored, particularly within real-world inpatient populations.

Age-sensitive mental health care is currently most clearly articulated for particular stages of the adult life course. Canadian guidance for emerging adults recommends a developmentally responsive continuum of care based on individuals’ changing needs rather than rigid age-based service thresholds [9]. Guidance for later-life mental health care similarly emphasizes integrated, recovery-oriented coordination among community services, primary care, general mental health, and specialized services [10]. More broadly, a life-course approach recognizes that developmental, functional, and social needs change throughout adulthood and should be considered in service planning [11]. Nevertheless, specific guidance for age-responsive psychiatric discharge planning across the full adult age range remains limited, supporting the need to examine age-related differences at this important transition in care.

In the Canadian context, psychiatric inpatient populations represent a clinically complex and heterogeneous group. According to the Canadian Institute for Health Information (CIHI), individuals hospitalized for mental health and substance use disorders experience some of the highest rates of early readmission following discharge [12]. These patterns highlight the importance of identifying baseline factors that may influence postdischarge trajectories. However, most existing studies focus on postdischarge outcomes or intervention effects, with relatively limited attention to how demographic characteristics such as age are associated with mental health status at the time of inpatient recruitment.

In Alberta, Canada, the Text4Support program was developed to address gaps in community-based mental health support through the provision of daily supportive text messages, alongside complementary peer support initiatives [13]. As part of the broader Text4Support research program, baseline data were collected from psychiatric inpatients at the time of recruitment, prior to the initiation of any intervention. This dataset provides a unique opportunity to examine age-related differences in mental health symptom severity within a real-world inpatient cohort at a critical transition point in care. Although the current analysis does not examine intervention effects, characterizing age-related differences at discharge provides important context for interpreting subsequent findings from the parent trial and may identify age-related needs to consider when developing and evaluating postdischarge supports.

The present study uses baseline data from the Text4Support project to examine the relationship between age and multiple mental health outcomes among psychiatric inpatients at the time of recruitment. Using validated instruments, including the Generalized Anxiety Disorder 7-item scale (GAD-7), the Patient Health Questionnaire-9 (PHQ-9), the World Health Organization-Five Well-Being Index (WHO-5), the Brief Resilience Scale (BRS), and the EQ-5D-5L, this study aims to assess age-related differences in anxiety, depression, overall well-being, resilience, and quality of life. By characterizing baseline mental health symptom profiles across age groups, this research seeks to inform the development of age-sensitive interventions and contribute to a more nuanced understanding of patient needs during the transition from inpatient to community-based mental health care. The analysis was intended to provide a descriptive, age-stratified profile of baseline mental health status rather than to estimate an independent causal effect of age or develop a multivariable prediction model.

## Methods

### Study Setting and Design

This study draws on data collected during the initial phase of an innovative program designed to enhance support and care for individuals discharged from inpatient mental health using e-mental health tools and peer worker services. The broader program adopted a pragmatic stepped-wedge cluster-randomized design and was implemented across 10 major health care sites in Alberta, located in Edmonton, Calgary, and Grande Prairie, between March 8, 2022, and February 29, 2024 [13]. Its purpose was to evaluate the effectiveness of postdischarge interventions, specifically supportive text messaging (Text4Support) alone or in combination with peer support, compared with treatment as usual [13]. This study involved secondary analysis of baseline data from the stepped-wedge trial and examines associations between age and multiple mental health measures among program participants. The present secondary analysis is restricted to baseline assessments completed before the delivery of Text4Support or peer support and therefore does not evaluate the effects of either intervention.

### Data Collection and Inclusion Criteria

A face-to-face recruitment process was implemented with nurse managers and clinical staff assisting the research team by identifying potential participants who were nearing discharge from inpatient psychiatric units. Eligible individuals were provided with detailed study information, including an information leaflet. Those who agreed to participate signed a paper-based consent form and completed a self-administered questionnaire on a tablet device. The survey was developed in REDCap (Vanderbilt University), a secure, browser-based platform for creating and managing online surveys and research databases, with support from members of the research team [14]. Data collected included sociodemographic characteristics (eg, age, gender, ethnicity, relationship status, employment status, education level, and housing status) and clinical information (eg, discharge diagnosis, anxiety, depression, and well-being levels). All enrolled participants were included in the analysis.

Participants were required to have a diagnosed mental health condition, be preparing for discharge from an inpatient psychiatric unit, be 18 years of age or older, and own a mobile device with an active phone number. They also needed the ability to receive and read English-language text messages and to provide written informed consent. Individuals planning to travel outside the city during the 6-month follow-up period were excluded due to the in-person nature of the peer support intervention. Phone numbers and health care numbers were collected as primary identifiers.

### Ethical Considerations

Ethical approval was obtained from the University of Alberta Health Research Ethics Board (reference number: Pro00111459), with additional operational approval granted by the regional health authority. Written informed consent was obtained from all participants.

### Sample Size Calculation

With 3 age clusters, a 2-tailed α level of 0.05% and 80% power (corresponding to a Type II error probability, β=0.20), and assuming a moderate effect size corresponding to Cohen *f*=0.25 (approximately equivalent to a pairwise Cohen *d* of 0.5 with a common SD of 1.0), we estimated that a total sample size of 159 participants (53 per age cluster) would be required to detect statistically significant differences between groups using a 1-way ANOVA [15].

### Outcome Measures

#### GAD-7

The GAD-7 was used to assess participants’ anxiety symptoms. This self-report measure consists of 7 items evaluating the frequency and severity of anxiety symptoms experienced over the preceding 2 weeks. Each item is rated on a 4-point Likert scale from 0 (“not at all”) to 3 (“nearly every day”), producing a total score ranging from 0 to 21, with higher scores indicating more severe anxiety [16]. The GAD-7 is widely applied in both clinical and research settings due to its strong psychometric properties. Using the recommended cutoff score of 10, the scale demonstrates a sensitivity of 89% and a specificity of 82% for detecting generalized anxiety disorder. It also shows strong test-retest reliability (intraclass correlation coefficient=0.83) and excellent internal consistency (Cronbach α=0.92) [16,17].

#### PHQ-9

The PHQ-9 was administered to assess depressive symptoms. Derived from the 9 *DSM-IV (Diagnostic and Statistical Manual of Mental Disorders, Fourth Edition)* diagnostic criteria for major depressive disorder, this self-administered tool measures the frequency of depressive symptoms over the past 2 weeks. Items are rated from 0 (“Never”) to 3 (“Nearly every day”), yielding total scores between 0 and 27 [18,19]. The PHQ-9 functions both as a diagnostic aid for major depressive disorder and as a measure of symptom severity. A score of 10 or above is commonly recommended to indicate clinically significant depression. Severity categories include mild (5), moderate (10), moderately severe (15), and severe (20). The scale has demonstrated good internal consistency (Cronbach α=0.85) and strong convergent validity, showing moderate-to-high correlations with measures such as the WHO-5, Hospital Anxiety and Depression Scale (HADS)–depression, HADS-anxiety, and GAD-7. At a cutoff of ≥10, sensitivity and specificity are both approximately 88% [18-20].

Item 3 of the PHQ-9 (“PHQ sleep”) [21,22] and item 9 (suicidal ideation) [23] provide specific indicators for sleep disturbance and suicide risk, respectively. The sleep item assesses difficulties falling asleep, staying asleep, or oversleeping in the prior 2 weeks. Research in primary care settings supports its effectiveness as a brief yet reliable screening tool for sleep problems without adding substantial response burden [21,22,24]. For instance, MacGregor et al [24] reported strong correlations between PHQ sleep scores and the Insomnia Severity Index, with a score of 1 offering an optimal balance between sensitivity and specificity.

PHQ-9 item 9 assesses suicidal ideation (“Thoughts that you would be better off dead or hurting yourself in some way”) and serves as a significant predictor of suicide risk independent of overall depression severity [23]. Higher scores on this item have been associated with markedly increased risk of suicide attempts and deaths for up to 2 years following assessment [25]. The item’s sensitivity (0.69) and specificity (0.84) further support its value as a routine screening indicator for suicide risk in primary care, facilitating earlier intervention for individuals who might otherwise go unrecognized [26].

#### WHO-5

The WHO-5 was used to evaluate participants’ overall mental well-being. This brief, positively worded self-report measure comprises 5 items reflecting subjective well-being, such as “I have felt cheerful and in good spirits,” “I have felt calm and relaxed,” and “My daily life has been filled with things that interest me” [27]. Each item is rated on a 6-point Likert scale from 0 (“not at all”) to 5 (“all the time”), and raw scores are converted to a standardized scale ranging from 0 (lowest well-being) to 100 (highest well-being). Scores below 50 indicate reduced well-being and suggest the need for further clinical assessment. The WHO-5 demonstrates strong internal consistency (Cronbach α=0.90) and good convergent validity, including a significant negative correlation with the PHQ-9 (*r*=−0.73; *P*<.001).

#### BRS

The BRS was used to measure participants’ resilience [28]. The scale contains 6 items, 3 positively worded and 3 negatively worded, rated on a 5-point Likert scale from 1 to 5, resulting in total scores ranging from 6 to 30 [29]. The BRS has acceptable internal consistency (α=0.66) and test-retest reliability (*r*=0.67) [29]. A respondent’s final score is calculated by dividing the total score by the number of completed items, with higher values indicating greater resilience.

Previous research classifies individual BRS mean item scores from 1.00 to 2.99 as low resilience, 3.00 to 4.30 as normal resilience, and 4.31 to 5.00 as high resilience [28,29]. These values represent individual-level classification thresholds rather than group means; therefore, SDs are not applicable. In the present study, the continuous BRS total score was used in analyses comparing resilience levels across age groups. For categorical analyses, individual mean item scores in the normal and high ranges (3.00‐5.00) were combined into a single category and compared with scores in the low-resilience range (1.00‐2.99).

#### EQ-5D-5L

The EQ-5D-5L, a widely used preference-based measure of health-related quality of life, was also administered. The instrument includes a descriptive system comprising 5 health dimensions: mobility, self-care, usual activities, pain/discomfort, and anxiety/depression [30,31]. Each dimension has 5 response levels ranging from 1 (no problems) to 5 (extreme problems). For this study, responses were dichotomized into 2 categories: “No Problems” (level 1) and “Problems” (levels 2‐5). The EQ-5D-5L is recognized for its reliability and validity in describing health status across diverse populations, with test-retest reliability reported between 0.65 and 0.91 [32,33].

### Statistical Analysis

All statistical analyses were conducted using SPSS software (version 25; IBM Corp) [34]. Descriptive statistics were used to summarize participants’ sociodemographic and clinical characteristics by age group, with categorical variables presented as frequencies and percentages and continuous variables reported as means and SDs.

Chi-square tests were performed to examine associations between age groups and categorized mental health measures, assessing whether the distributions of these indicators differed significantly across age groups.

Age was categorized as 18 to 25, 26 to 40, and >40 years. The 18- to 25-year category was selected to represent emerging adulthood, a developmental period characterized by major transitions in education, employment, relationships, and independent living [35,36]. The remaining cut points were selected pragmatically to permit comparisons across subsequent adult age ranges while maintaining adequate sample sizes in each group. These categories are study-defined and do not represent established clinical thresholds. In particular, the older than 40 years category includes both middle-aged and older participants and was therefore not interpreted as a uniform older-adult group. For continuous mental health outcomes, including anxiety, depression, sleep disturbance, suicidal ideation, resilience, well-being, and health-related quality of life, 1-way ANOVA was conducted to compare mean scores across age groups. When the assumption of homogeneity of variance was violated, Welch ANOVA was applied. For outcomes demonstrating significant overall group differences, post hoc pairwise comparisons were conducted using Tukey HSD and Games-Howell tests to identify specific age-group differences. The analyses were designed as unadjusted, descriptive comparisons across age groups. Education level, employment status, and discharge diagnosis were summarized by age group to characterize the sample but were not entered as covariates because independent predictor modeling was outside the scope of the present analysis.

## Results

### Baseline Data Analysis

Participants ranged in age from 18 to 81 years, with a mean age of 34.06 years and an SD of 14.05. Of the 1106 participants, 403 (36.4%) were aged 18 to 25 years, 378 (34.2%) were aged 26 to 40 years, and 325 (29.4%) were over 40 years of age. Among respondents, 468 (42.3%) identified as male, 606 (54.8%) as female, and 32 (2.9%) as another gender. The majority (n=681, 61.6%) identified as Caucasian. More than half (n=568, 51.4%) reported completing a high school education, 652 (59.0%) were single, and 585 (52.9%) were unemployed. In terms of living arrangements, 459 (41.5%) participants lived with family or friends. A total of 294 (26.6%) respondents reported having a diagnosis of depression. A detailed summary of participant characteristics is presented in Table 1.

**Table 1.** Distribution of sociodemographic and clinical characteristics among the study participants.

Variables	Age groups (years), n (%)			
	18-25 (n=403)	26-40 (n=378)	>40 (n=325)	Total (N=1106)
Gender				
Male	157 (39.0)	168 (44.4)	143 (44.0)	468 (42.3)
Female	226 (56.1)	199 (52.6)	181 (55.7)	606 (54.8)
Other	20 (5.0)	11 (2.9)	1 (0.3)	32 (2.9)
Ethnicity				
Caucasian	210 (52.1)	235 (62.2)	236 (72.6)	681 (61.6)
Indigenous	43 (10.7)	31 (8.2)	28 (8.6)	102 (9.2)
Black	63 (15.6)	41 (10.8)	16 (4.9)	120 (10.8)
Asian	60 (14.9)	40 (10.6)	25 (7.7)	125 (11.3)
Mixed/other	27 (6.7)	31 (8.2)	20 (6.2)	78 (7.1)
Education level				
Less than high school	15 (3.7)	16 (4.2)	10 (3.1)	41 (3.7)
High school diploma	297 (73.7)	161 (42.6)	110 (33.8)	568 (51.4)
Postsecondary education	77 (19.1)	196 (51.9)	190 (58.5)	463 (41.9)
Prefer not to say	14 (3.5)	5 (1.3)	15 (4.6)	34 (3.1)
Relationship status				
Married/partnered/common-law	75 (18.6)	110 (29.1)	138 (42.5)	323 (29.2)
Single	306 (75.9)	237 (62.7)	109 (33.5)	652 (59.0)
Separated or divorced	0 (0)	24 (6.3)	63 (19.4)	87 (7.9)
Widowed	0 (0)	3 (0.8)	9 (2.8)	12 (1.1)
Prefer not to say	22 (5.5)	4 (1.1)	6 (1.8)	32 (2.9)
Employment status				
Employed	91 (22.6)	134 (35.4)	103 (31.7)	328 (29.7)
Unemployed	215 (53.3)	222 (58.7)	148 (45.5)	585 (52.9)
Student	78 (19.4)	10 (2.6)	1 (0.3)	89 (8.0)
Retired	0 (0)	4 (1.1)	64 (19.7)	68 (6.1)
Other	19 (4.7)	8 (2.1)	9 (2.8)	36 (3.3)
Housing status				
Own home	16 (4.0)	64 (16.9)	141 (43.1)	221 (20.0)
Rented accommodation	82 (20.3)	149 (39.4)	127 (39.1)	358 (32.4)
Live with family or friend	285 (70.7)	139 (36.8)	35 (10.8)	459 (41.5)
Couch surfing/shelter/street/other	20 (5.0)	26 (6.9)	22 (6.8)	68 (6.1)
Primary mental health diagnosis				
Depression	99 (24.6)	84 (22.2)	111 (34.2)	294 (26.6)
Bipolar disorder	68 (16.9)	89 (23.5)	66 (20.3)	223 (20.2)
Anxiety	56 (13.9)	40 (10.6)	47 (14.5)	143 (12.9)
Schizophrenia	63 (15.6)	80 (21.2)	35 (10.8)	178 (16.1)
Personality disorder	70 (17.4)	34 (9.0)	3 (0.9)	107 (9.7)
Substance use disorder	4 (1.0)	4 (1.1)	7 (2.2)	15 (1.4)
Other	43 (10.7)	47 (12.4)	56 (17.2)	146 (13.2)

### Chi-Squared Analysis

Tables 2 and 3 present the chi-square analyses examining baseline clinical measures across 3 age groups (18‐25, 26‐40, and >40 years). Several mental health indicators showed significant variation by age. Resilience, as measured using the BRS, differed significantly across groups (*P*=.04). Several categorical mental health outcomes differed significantly across age groups. Low resilience was reported by 60.8% (n=245) of participants aged 18 to 25 years, compared with 52.6% (n=199) of those aged 26 to 40 years and 53.4% (n=173) of those aged older than 40 years (*χ*²_2_=6.36; *P*=.04). Poor well-being was reported by 52.5% (n=211), 42.9% (n=162), and 50.9% (n=165) of the 3 groups, respectively (*χ*²_2_=8.12; *P*=.02).

**Table 2.** Clinical baseline measures by age group (BRS^a^, WHO-5^b^, PHQ-9^c^, and suicidal ideation).

Variables	Age groups (years), n (%)			*χ*^2^ (*df*)	*P* value
	18-25^d^	26-40	>40		
BRS				6.36 (2)	.04^e^
Normal-high resilience	158 (39.2)	179 (47.4)	151 (46.6)		
Low resilience	245 (60.8)	199 (52.6)	173 (53.4)		
WHO-5				8.12 (2)	.02^e^
Good well-being	191 (47.5)	216 (57.1)	159 (49.1)		
Poor well-being	211 (52.5)	162 (42.9)	165 (50.9)		
PHQ-9				6.81 (2)	.03^e^
Unlikely depression	153 (38.1)	169 (44.7)	153 (47.2)		
Likely depression	249 (61.9)	209 (55.3)	171 (52.8)		
Sleep issues				4.49 (2)	.11
No sleep issues	70 (17.4)	78 (20.6)	77 (23.8)		
With sleep issues	332 (82.6)	300 (79.4)	247 (76.2)		
Suicidal ideation				19.09 (2)	<.001^e^
No	169 (42.0)	210 (55.6)	181 (55.9)		
Yes	233 (58.0)	168 (44.4)	143 (44.1)		
GAD-7^f^				3.57 (2)	.17
Unlikely anxiety	246 (61.3)	234 (62.1)	216 (67.7)		
Likely anxiety	155 (38.7)	143 (37.9)	103 (32.3)		

a BRS: Brief Resilience Scale.

b WHO-5: World Health Organization-Five Well-Being Index.

c PHQ-9: Patient Health Questionnaire-9.

d Percentages are based on available responses. Measure-specific denominators for the 18- to 25-, 26- to 40-, and >40-year groups, respectively, were as follows: BRS: n=403, 378, and 324; WHO-5, PHQ-9, sleep issues, and suicidal ideation: n=402, 378, and 324; and GAD-7: n=401, 377, and 319.

e Significant *P* value.

f GAD-7: Generalized Anxiety Disorder 7-item scale.

**Table 3.** Clinical baseline measures by age group (EQ-5D-5L).

Variable	Age groups (years), n (%)			*χ*^2^ (*df*)	*P* value
	18-25^a^	26-40	>40		
Mobility				22.65 (2)	<.001^b^
No problems	318 (79.3)	298 (79.0)	209 (65.5)		
Problems present	83 (20.7)	79 (21.0)	110 (34.5)		
Self-care				4.47 (2)	.11
No problems	310 (77.3)	307 (81.4)	239 (74.9)		
Problems present	91 (22.7)	70 (18.6)	80 (25.1)		
Usual activity				5.30 (2)	.07
No problems	213 (53.1)	208 (55.2)	149 (46.7)		
Problems present	188 (46.9)	169 (44.8)	170 (53.3)		
Anxiety/depression				12.63 (2)	<.001^b^
No problems	75 (18.7)	108 (28.6)	89 (27.9)		
Problems present	326 (81.3)	269 (71.4)	230 (72.1)		
Pain/discomfort				4.14 (2)	.13
No	169 (42.1)	173 (45.9)	122 (38.2)		
Yes	232 (57.9)	204 (54.1)	197 (61.8)		

a Percentages are based on available responses. The measure-specific denominators for the 18- to 25-, 26- to 40-, and >40-year groups were n=401, n=377, and n=319, respectively.

b Significant *P* value.

The proportion screening positive for probable depression was 61.9% (n=249) among participants aged 18 to 25 years, 55.3% (n=209) among those aged 26 to 40 years, and 52.8% (n=171) among those aged older than 40 years (*χ*²_2_=6.81; *P*=.03). Suicidal ideation, assessed using PHQ-9 item 9, was reported by 58.0%, 44.4%, and 44.1% of the 3 groups, respectively (*χ*²_2_=19.09; *P*<.001). No statistically significant age-group differences were observed in categorical sleep difficulties or anxiety.

For the EQ-5D-5L dimensions, mobility problems were reported by 20.7% (n=83) of participants aged 18 to 25 years, 21.0% (n=79) of those aged 26 to 40 years, and 34.5% (n=110) of those aged older than 40 years (*χ*²_2_=22.65, *P*<.001). Problems in the anxiety/depression dimension were reported by 81.3% (n=326), 71.4% (n=269), and 72.1% (n=230) of the 3 groups, respectively (*χ*²_2_=12.63, *P*<.001). No statistically significant age-group differences were found for self-care, usual activities, or pain/discomfort.

### ANOVA Analysis

One-way ANOVA was conducted to examine age-group differences in continuous baseline mental health measures across 3 age categories (18‐25 y, 26‐40 y, and >40 y; Table 4). When the assumption of homogeneity of variance was violated, Welch ANOVA was applied. For outcomes showing significant omnibus effects, post hoc pairwise comparisons were conducted using Games-Howell tests to identify specific age-group differences (Table 5). Consistent with the chi-square analyses of categorized outcomes, significant age-related differences were observed for resilience, depressive symptoms, anxiety symptoms, suicidal ideation, and recovery, with younger participants generally reporting poorer mental health outcomes.

**Table 4.** ANOVA test parameters for different age groups.

Variable	Age groups (years), mean (SD)			*F* test (*df*)	*P* value
	18-25	26-40	>40		
BRS^a^	15.96 (4.67)	17.34 (5.14)	17.09 (4.90)	8.73 (2,1102)	<.001^b^
WHO-5^c^	49.63 (22.85)	53.31 (22.38)	51.11 (24.39)	2.48 (2,1101)	.08
PHQ-9^d^	12.12 (6.33)	11.03 (6.70)	10.46 (6.56)	6.17 (2,1101)	<.001^b^
Suicidal ideation	0.94 (1.01)	0.69 (0.92)	0.68 (0.92)	9.53 (2,1101)	<.001^b^
Sleep issues	1.50 (0.99)	1.46 (1.03)	1.43 (1.07)	.41 (2,1101)	.67
GAD-7^e^	9.02 (5.38)	8.71 (6.01)	7.44 (5.35)	7.74 (2,1094)	<.001^b^
EQ-5D-5L	87.69 (14.87)	92.08 (15.16)	90.11 (16.01)	8.07 (2,1100)	<.001^b^

a BRS: Brief Resilience Scale.

b Significant *P* value.

c WHO-5: World Health Organization-Five Well-Being Index.

d PHQ-9: Patient Health Questionnaire-9.

e GAD-7: Generalized Anxiety Disorder 7-item scale.

**Table 5.** Pairwise comparison of the baseline mean scores on the scales for participants of different age groups.

Variable, study group, and paired groups	Mean difference (SE)	*P* value	95% CI
BRS^a^ (y)			
Study group: 18-25			
Paired group: 26-40	−1.38 (0.35)	<.001^b^	−2.20 to −0.55
Paired group: >40	−1.13 (0.36)	.01^b^	−1.97 to 0.29
Study group: 26-40			
Paired group: 18-25	1.38 (0.35)	<.001^b^	0.55 to 2.20
Paired group: >40	0.25 (0.38)	.79	−0.64 to 1.14
Study group: >40			
Paired group: 18-25	1.13 (0.36)	.01^b^	0.29 to 1.97
Paired group: 26-40	−0.25 (0.38)	.79	−1.14 to 0.64
PHQ-9^c^ (y)			
Study group: 18-25			
Paired group: 26-40	1.08 (0.47)	.05	−0.01 to 2.18
Paired group: >40	1.66 (0.48)	<.001^b^	0.53 to 2.79
Study group: 26-40			
Paired group: 18-25	−1.08 (0.47)	.05	−2.18 to 0.01
Paired group: >40	0.58 (0.50)	.48	−0.60 to 1.76
Study group: >40			
Paired group: 18-25	−1.66 (0.48)	<.001^b^	−2.79 to −.053
Paired group: 26-40	−0.58 (0.50)	.48	−1.76 to 0.60
Suicidal Ideation (y)			
Study group: 18-25			
Paired group: 26-40	0.26 (0.07)	<.001^b^	0.10 to 0.42
Paired group: >40	0.26 (0.07)	<.001^b^	0.10 to 0.43
Study group: 26-40			
Paired group: 18-25	−0.26 (0.07)	<.001^b^	−0.42 to −0.10
Paired group: >40	0.01 (0.07)	>.99	−0.16 to 0.17
Study group: >40			
Paired group: 18-25	−0.26 (0.07)	<.001^b^	−0.43 to −0.10
Paired group: 26-40	−0.01 (0.07)	>.99	−0.17 to 0.16
GAD-7^d^ (y)			
Study group: 18-25			
Paired group: 26-40	0.31 (0.41)	.72	−0.64 to 1.28
Paired group: >40	1.59 (0.41)	<.001^b^	0.64 to 2.53
Study group: 26-40			
Paired group: 18-25	−0.31 (0.41)	.72	−1.28 to 0.65
Paired group: >40	1.27 (0.43)	.01	0.26 to 2.29
Study group: >40			
Paired group: 18-25	−1.59 (0.40)	<.001^b^	−2.53 to −0.64
Paired group: 26-40	1.27 (0.43)	.01	−2.28 to −0.26
EQ-5D-5L (y)			
Study group: 18-25			
Paired group: 26-40	−4.40 (1.08)	<.001^b^	−6.92 to −1.87
Paired group: >40	−2.42 (1.16)	.09	−5.15 to 0.30
Study group: 26-40			
Paired group: 18-25	4.40 (1.08)	<.001^b^	1.87 to 6.92
Paired group: >40	1.97 (1.18)	.22	−0.91 to 4.75
Study group: >40			
Paired group: 18-25	2.42 (1.16)	.09	−0.30 to 5.15
Paired group: 26-40	−1.97 (1.18)	.22	−4.76 to 0.81

a BRS: Brief Resilience Scale.

b Significant *P *value.

c PHQ-9: Patient Health Questionnaire-9.

d GAD-7: Generalized Anxiety Disorder 7-item scale.

Resilience, assessed using the BRS, differed significantly across age groups (Welch *F*_2, 717.35_=8.98, *P*<.001), with a small effect size (η²≈0.02). Post hoc Games-Howell comparisons indicated that participants aged 18 to 25 years reported significantly lower resilience than those aged 26 to 40 years (*P*<.001) and those over 40 years of age (*P*<.05); in contrast, no significant difference was observed between the 2 older age groups.

Depressive symptom severity, measured by the PHQ-9, also varied significantly by age (Welch *F*_2, 718.19_=6.34, *P*=.002), with a small effect size (η²≈0.01). Games-Howell post hoc tests revealed that participants aged 18 to 25 years reported significantly higher depression scores than those over 40 years of age (*P*<.001), aligning with the higher prevalence of likely depression observed in the youngest age group in the categorical analyses.

Anxiety severity, assessed using the GAD-7, demonstrated significant age-group differences (Welch *F*_2, 715.12_=8.33, *P*<.001), with a small-to-moderate effect size (η²≈0.02). Post hoc analyses showed that both the 18 to 25 year and 26 to 40 year groups reported significantly higher anxiety scores compared with participants over 40 years of age (*P*<.001 and *P*=.01, respectively), consistent with the chi-square findings, indicating higher rates of anxiety among younger participants.

Suicidal ideation, measured using item 9 of the PHQ-9, also differed significantly across age groups (Welch *F*_2, 725.35_=9.05; *P*<.001), with a small-to-moderate effect size (η²≈0.02). Games-Howell post hoc comparisons indicated that participants aged 18 to 25 years reported significantly higher levels of suicidal ideation than both the 26 to 40 years and older than 40 years groups (both *P*<.001), with no significant difference between the 2 older groups.

Recovery scores showed significant age-related variation (Welch *F*_2, 713.12_=8.38, *P*<.001), with a small effect size (η²≈0.01). Post hoc analyses revealed that participants aged 18 to 25 years reported significantly lower recovery scores than those aged 26 to 40 years (*P*<.001), mirroring the lower resilience and poorer mental health profiles observed among younger participants.

Quality of life, measured using the EQ-5D-5L index score, also differed significantly across age groups (*F*_2, 1100_=8.07, *P*<.001), with a small effect size. Participants aged 18 to 25 years reported significantly lower EQ-5D-5L scores compared with those aged 26 to 40 years, indicating poorer overall perceived health status among younger participants, while scores among participants aged over 40 years fell between the 2 younger age groups.

No significant age-group differences were observed for overall well-being as measured by the WHO-5 (Welch *F*_2, 714.09_=2.60, *P*=.08), or for sleep disturbance assessed using the PHQ-9 sleep item (Welch *F*_2, 714.35_=0.41, *P*=.67).

## Discussion

### Principal Findings

This study examined age-related differences in baseline demographic, clinical, and mental health characteristics among individuals enrolled in the Text4Support program following discharge from inpatient psychiatric care. By analyzing both categorized and continuous mental health measures, the findings provide a nuanced understanding of how age is associated with mental health vulnerability at the point of discharge, a critical transitional period. Overall, the results indicate that younger adults, particularly those aged 18 to 25 years, experience disproportionately higher levels of psychological distress, lower resilience, and poorer recovery-related outcomes compared with older age groups.

Baseline analyses revealed a high prevalence of mental health symptoms across the entire sample, underscoring the substantial burden of psychiatric distress at hospital discharge. Rates of depression, anxiety, poor well-being, and suicidal ideation were considerable, reinforcing previous evidence that the postdischarge period represents a time of heightened vulnerability [37-39]. These findings align with broader literature highlighting increased risks of relapse, emergency service use, and mortality following psychiatric discharge, particularly when continuity of care is limited. The substantial symptom burden observed among participants preparing for discharge warrants careful clinical interpretation. Screening positive for depression or endorsing PHQ-9 item 9 does not, by itself, establish that discharge was inappropriate or that inpatient treatment was inadequate. Discharge decisions are informed by comprehensive clinical assessment, including symptom change, functional status, immediate risk, treatment response, available supports, and the feasibility of community follow-up. Moreover, PHQ-9 item 9 is a screening indicator of thoughts of death or self-harm and should not be interpreted as a stand-alone assessment of imminent suicide risk [40-42]. Because this study did not examine inpatient treatment processes, changes during hospitalization, or the clinical basis for discharge decisions, conclusions regarding the quality of acute inpatient care cannot be drawn.

Age-stratified analyses demonstrated consistent and meaningful differences across several mental health indicators. Younger participants reported significantly higher levels of depressive and anxiety symptoms, greater suicidal ideation, and lower resilience and recovery scores compared with participants aged over 40 years. These patterns were evident in both chi-square analyses of categorized outcomes and Welch ANOVA analyses of continuous measures, lending robustness to the findings. The convergence of findings across analytical approaches indicates that age-group differences were present across several mental health measures. However, because the analyses were unadjusted, these differences may also reflect variation in education, employment, diagnosis, or other sociodemographic and clinical characteristics.

The heightened vulnerability observed among individuals aged 18 to 25 years may be explained by a combination of developmental, social, and structural factors [35,36]. Young adulthood is a period marked by ongoing identity formation, emotional regulation development, and life transitions related to education, employment, and relationships. Psychiatric hospitalization during this stage may disrupt these processes and compound existing stressors [35]. In addition, younger adults in this sample were more likely to report low resilience and poorer recovery, which may reflect fewer established coping strategies or limited access to stable social and economic support. These findings are consistent with previous research demonstrating that younger psychiatric patients often experience more severe symptomatology and poorer psychosocial functioning at discharge compared with older cohorts [35,36].

In contrast, participants aged over 40 years generally demonstrated lower levels of depression and anxiety, fewer reports of suicidal ideation, and higher resilience and recovery scores. While older adults may face their own set of challenges, including chronic illness or social isolation, they may also benefit from accumulated coping skills, greater emotional regulation, or more stable life circumstances [43]. The observed age gradient in mental health outcomes highlights the importance of considering age as a key factor in postdischarge risk stratification and intervention planning.

Quality-of-life analyses further supported these age-related differences. Participants aged older than 40 years reported a higher prevalence of mobility problems, reflecting age-related physical health concerns, whereas younger participants reported substantially higher levels of anxiety and depression within the EQ-5D-5L framework. This distinction underscores the need for age-sensitive approaches to postdischarge care that address not only psychiatric symptoms but also broader functional and quality-of-life domains relevant to different age groups [44]. Viewed in relation to existing service frameworks, the greater psychological distress observed among participants aged 18 to 25 years supports calls for developmentally responsive follow-up and stronger continuity of care for emerging adults [9]. The higher prevalence of mobility problems among participants aged older than 40 years also indicates that discharge planning should consider functional and physical health needs alongside psychiatric symptoms. However, because the older than 40 years category included both middle-aged and older participants, this finding should not be interpreted as evidence specific to geriatric mental health services.

Notably, no significant age-group differences were observed for overall well-being measured by the WHO-5 or for sleep disturbance when analyzed as continuous outcomes. These findings may suggest that certain aspects of subjective well-being and sleep difficulties are pervasive across age groups at discharge, potentially reflecting the shared impact of acute psychiatric hospitalization. Alternatively, these measures may be less sensitive to age-related variation at baseline, warranting further investigation in longitudinal analyses.

Although this baseline analysis did not evaluate Text4Support, the observed age-related patterns may inform hypotheses for subsequent intervention analyses, including whether engagement or outcomes vary across age groups. In particular, the greater psychological distress and lower resilience observed among participants aged 18 to 25 years may be relevant when considering the content, intensity, and delivery of postdischarge support. However, the present findings do not establish that Text4Support is effective or that any age-specific adaptation would improve outcomes.

From a service delivery perspective, these findings support incorporating both symptom severity and age-specific considerations into discharge planning and transitional care. Patients with persistent symptoms may benefit from comprehensive clinical and suicide risk assessment before discharge, collaborative safety planning, medication reconciliation, a written crisis plan, and confirmed follow-up arrangements [38,42,45]. A warm handover to community providers and proactive postdischarge contact may reduce gaps in care, particularly when suicide risk has been identified [42,46,47]. Younger patients may benefit from more structured follow-up, proactive engagement, and interventions targeting resilience and recovery skills, while participants aged older than 40 years may require additional attention to functional limitations and physical health concerns. Continued symptom monitoring can support early identification of deterioration; however, supportive text messaging should be considered an adjunct to, rather than a replacement for, timely clinical follow-up and continuing care [46-50].

### Limitations

Several limitations should be acknowledged. First, this cross-sectional analysis was based on baseline data, precluding causal inference and conclusions about symptom trajectories over time. It also cannot determine the effectiveness, accessibility, or acceptability of Text4Support, whether engagement with the intervention differs by age, or whether age modifies intervention response. Second, the mental health outcomes were assessed using self-report instruments and may therefore be affected by reporting bias. Third, the older than 40 years category encompassed a broad age range and included participants at different stages of adulthood. Combining middle-aged and older participants may have obscured age-specific patterns within this group; consequently, the findings should not be interpreted as representing a distinct older-adult population. Finally, education level, employment status, and discharge diagnosis were reported descriptively in Table 1 but were not examined jointly in relation to the mental health outcomes. The reported age-group comparisons are therefore unadjusted and cannot be interpreted as effects of age independent of these sociodemographic and clinical characteristics. Other factors not included in the present analysis, including broader socioeconomic circumstances and social support, may also influence mental health status at discharge. Future hypothesis-driven analyses should examine these characteristics and their potential interactions with age.

Despite these limitations, this study provides valuable insight into age-related disparities in mental health status at psychiatric discharge within a large, real-world sample. The findings underscore the need for age-informed transitional care strategies and support the integration of digital mental health interventions within broader, flexible care pathways. Future research should examine how age interacts with intervention engagement and outcomes over time and whether tailored approaches can mitigate the elevated risks observed among younger adults during this critical period.

### Conclusions

This study demonstrates significant age-related differences in baseline mental health and quality-of-life outcomes among individuals discharged from psychiatric inpatient care and enrolled in the Text4Support program. Younger adults, particularly those aged 18 to 25 years, exhibited higher levels of psychological distress, lower resilience, and poorer recovery-related outcomes compared with participants aged older than 40 years, underscoring their heightened vulnerability during the transition to community care.

The findings emphasize the importance of incorporating age as a key consideration in postdischarge assessment and planning. Participants aged 18 to 25 years with elevated baseline risk profiles may require more tailored or intensified transitional care, whereas participants aged older than 40 years may benefit from supports that address functional limitations alongside mental health needs.

These baseline findings provide context for future evaluation of Text4Support and other postdischarge supports, including whether intervention engagement and outcomes vary across age groups. Integrating baseline risk stratification into transitional care planning may enhance the effectiveness of postdischarge supports and improve outcomes for vulnerable populations.

## Abbreviations

BRS: Brief Resilience Scale
CIHI: Canadian Institute for Health Information
*DSM-IV*: *Diagnostic and Statistical Manual of Mental Disorders*, *Fourth Edition*
GAD-7: Generalized Anxiety Disorder 7-item scale
HADS: Hospital Anxiety and Depression Scale
PHQ-9: Patient Health Questionnaire-9
WHO-5: World Health Organization-Five Well-Being Index
